# Scorpion Venom Analgesic Peptide, BmK AGAP Inhibits Stemness, and Epithelial-Mesenchymal Transition by Down-Regulating PTX3 in Breast Cancer

**DOI:** 10.3389/fonc.2019.00021

**Published:** 2019-01-25

**Authors:** Sylvanus Kampo, Bulbul Ahmmed, Tingting Zhou, Lawrence Owusu, Thomas Winsum Anabah, Natacha Raissa Doudou, Eugene Dogkotenge Kuugbee, Yong Cui, Zhili Lu, Qiu Yan, Qing-Ping Wen

**Affiliations:** ^1^Department of Anesthesiology, Dalian Medical University, Dalian, China; ^2^Department of Anesthesiology, First Affiliated Hospital of Dalian Medical University, Dalian, China; ^3^Department of Anesthesia and Intensive Care, School of Medicine and Health Science, University for Development Studies, Tamale, Ghana; ^4^Department of Biochemistry and Molecular Biology, Dalian Medical University, Dalian, China; ^5^Department of Biotechnology, Dalian Medical University, Dalian, China; ^6^Department of Radiology, Dalian Medical University, Dalian, China; ^7^Department of Clinical Microbiology, School of Medicine and Health Science, University for Development Studies, Tamale, Ghana; ^8^School of Life Science and Bio-pharmaceutics, Shenyang Pharmaceutical University, Shenyang, China; ^9^Department of Ophthalmology, First Affiliated Hospital of Dalian Medical University, Dalian, China

**Keywords:** scorpion venom analgesic peptide, rBmK AGAP, stemness, epithelial-mesenchymal transition, pentraxin 3, Wnt/β-catenin signaling, transcription factor NF-κB, breast cancer

## Abstract

A scorpion peptide reported to exhibit both analgesic and antitumor activity in animal models may present as an alternative therapeutic agent for breast cancer. We aimed to investigate the effect of *Buthus martensii Karsch* antitumor-analgesic peptide (BmK AGAP) on breast cancer cell stemness and epithelial-mesenchymal transition (EMT). We treated MCF-7 and MDA-MB-231 cells with different concentrations of rBmK AGAP and observed that rBmK AGAP inhibited cancer cell stemness, epithelial-mesenchymal transition (EMT), migration, and invasion. Analysis by qPCR, ELISA, western blot, immunofluorescence staining, sphere formation, colony assay, transwell migration, and invasion assays demonstrated rBmK AGAP treatment decreased the expressions of Oct4, Sox2, N-cadherin, Snail, and increased the expression of E-cadherin. rBmK AGAP inhibited breast cancer cell stemness, EMT, migration, and invasion by down-regulating PTX3 through NF-κB and Wnt/β-catenin signaling Pathway *in vitro* and *in vivo*. Xenograft tumor model confirmed inhibition of tumor growth, stem-like features, and EMT by rBmK AGAP. Thus, rBmK AGAP is a potential therapeutic agent against breast cancer and related pain.

## Introduction

Breast cancer is one of the leading cause of cancer-related morbidity and mortality among women worldwide with an estimated 25% of all newly diagnosed cancers being breast cancer annually (1). Invasion and metastasis are major causes of breast cancer-associated morbidity and mortality (2). Despite the existence of all-inclusive therapies (radiotherapy, chemotherapy, and radical surgery), the prognosis of metastatic breast cancer nevertheless remains uncertain (3). Though current conventional drugs have demonstrated promise in cancer therapy, effective, and target therapeutic approaches for breast cancer and related pain are still needed. Metastatic tumors are characterized by highly aggressive and angiogenic phenotype and extensive areas of necrosis surrounded by highly anaplastic cells. Necrotic cell death and hypoxia associated with tumor progression trigger the release of inflammatory mediators which play critical role in tumorigenesis and cancer-related pain (4). One such mediator is pentraxin 3 (PTX3), a member of the pentraxin superfamily. Aside inflammation, PTX3 functions in complements activation regulation, modification of angiogenesis, and tissue remodeling (5). Expression of PTX3 is rapidly induced in a variety of cell types by several stimuli including cytokines and NF-κB activation (6).

Pain is a major distressing symptom of cancer and one of the classical signs of inflammation during cancer progression. Opioid analgesics are the most frequently used analgesic for perioperative anesthetic and cancer-related pain. However, previous studies have indicated that morphine and fentanyl promote cancer cell stemness, epithelial-mesenchymal transition, and drug resistance (7, 8). Other studies have also indicated up-regulation of mu-opioid receptor in cancer cells to enhance primary tumor growth, metastasis and stemness (9, 10).

Cancer stem cells are heterogeneous tumor cells and play key roles in tumor initiation, metastasis, therapy resistance, and tumor recurrence. Breast cancer stem cells have the characteristic of unlimited self-renewal and the ability to generate differentiated descendants (11, 12). The regulatory mechanism of breast cancer cells' stemness remains elusive. They can quickly transform to mesenchymal phenotype in a process referred to as epithelial to mesenchymal transition (EMT). Epithelial cells possess a high level of plasticity. Epithelial-mesenchymal transition is often accompanied with increase in stemness (13, 14). This kind of mechanism is necessary for embryo development and is required for the acquisition of invasive properties by cancer (15, 16). Epithelial to mesenchymal transition is an early indication of cancer metastasis which is shown by the loss of cell-cell adhesion and polarity. This, in particular, often fosters cancer cell migration, invasion, and metastasis (17). During the process of epithelial-mesenchymal transition, reduced expression of intercellular adhesion protein E-cadherin and increased expression of N-cadherin, snail1, twist, ZEB1, ZEB2, and Vimentin occur (18, 19). E-cadherin is an essential epithelial-mesenchymal transition marker, and when interacting with catenins, it forms the E-cadherin/β-catenin/α-catenin complex linked to the actin cytoskeleton (20). Reduced expression of E-cadherin promotes intracellular junction damage, giving the epithelial cell the potential to migrate, thereby increasing invasion and metastasis (21). Emerging evidence shows that epithelial-mesenchymal transition plays a vital role during drug resistance development in cancer (22).

*Buthus martensii Karsch* (BmK) venom contains mixtures of peptides that have analgesic and antitumor activities (23). The first BmK analgesic peptide was purified from the venom in 1994 by Wang and colleagues (24). Since then, more BmK analgesic peptides including BmK AGAP have been purified for pain and cancer management (25). *Buthus martensii Karsch* venom and its extracts have been used for many decades in Asia and some parts of the world to treat cancer and pain. The scorpion, *Buthus martensii Karsch* analgesic peptide, BmK AGAP belongs to a group of long-chain scorpion peptides and has a molecular mass of 7142Da with 66 amino acid residues (26, 27). Reports have shown that BmK AGAP has both analgesic and antitumor properties. Many animal studies have demonstrated the analgesic activity of BmK AGAP (28–30). However, little is known about the antitumor activity of BmK AGAP, especially on cancer stemness and epithelial-mesenchymal transition. Hence, this study aimed to investigate the effects of BmK AGAP on cancer cell stemness and epithelial-mesenchymal transition of breast cancer cells.

## Materials and Methods

### Ethics Statement and Clinical Samples

The ethical committee of the First Affiliated Hospital of Dalian Medical University approved for collection and use of clinical samples. Thirty-six female patients diagnosed with first-grade (*n* = 12), second-grade (*n* = 13), or third-grade (*n* = 11) breast cancer and was confirmed by histopathology analysis and 42 normal female patients with no history of breast cancer who reported at the surgical unit for mastectomy or breast biopsy were recruited for this study after obtaining written informed consent between January 2017 and April 2018. The mean ages of the patients recruited were 53 and 36 years old for the breast cancer patients and the normal patients, respectively. All breast cancer paraffin sections and breast cancer tissues were obtained at the First Affiliated Hospital of the Dalian Medical University, China.

### Cell Culture

The human breast cancer cells MCF-10A, MCF-7, MDA-MB-231, and BT549, were purchased from the American Type Culture Collection (Beijing Zhongyuan limited, China). Using short tandem repeat (STR) analysis, the MCF-10A, MCF-7, MDA-MB-231, and BT549 cells were authenticated by Beijing Microread Genetics (Beijing, China) before purchase. The MCF-10A, MCF-7, MDA-MB-231, and BT549 cells were routinely maintained in DMEM/F12 or high-glucose DMEM (Gibco, USA) medium, supplemented with 10% fetal bovine serum (FBS) (Gibco, USA), penicillin 100 units/ml and streptomycin 100 μg/ml (TransGen Biotech, China). The cells were maintained in an incubator at 37°C humidified air with 5% CO_2_ atmospheric condition. The MCF-10A, MCF-7, MDA-MB-231, and BT549 cells were routinely subcultured every 3–5 days.

### Preparation of Recombinant BmK AGAP

Recombinant BmK AGAP (rBmK AGAP) was provided by Shenyang pharmaceutical University School of Life Science and Bio-pharmaceutics (Shenyang, China). The rBmK AGAP was obtained as described previously (27). The rBmK AGAP solution was diluted with 0.9% saline or PBS and filtered with a 0.22 μm sterile membrane before used. The activity of rBmK AGAP was the same as in the previous study.

### Antibodies and Reagents

The sources of antibodies and reagents were: PTX3 antibodies #13797-1-AP (proteintech, China); Oct4 antibodies # 11263-1-AP (proteintech, China); Sox2 antibodies #11064-1-AP (Proteintech, China); Nanog antibodies #14295-1-AP (proteintech, China); E-cadherin antibodies #20874-1-AP (proteintech, China); N-cadherin antibodies #22018-1-AP (Proteintech, China); Snai1 antibodies #13099-1-AP (proteintech, China); Vimentin antibodies #10366-1-AP (Proteintech, China); Nav 1.5 antibody #23016-1-AP (Proteintech, China); NF-κB antibodies (Selleck, USA); p65/NF-κB # 10745-1-AP and p-p65 antibodies (Proteintech, China); IKKα and IκBα antibodies (Selleck, USA); pGSK3-β antibodies (Abcam, USA); GSK3-β antibodies (Abcam, USA); β-catenin antibodies # 51067-2-AP (proteintech, China); TNF-α (Proteintech, China); Peroxidase-conjugated goat anti-rabbit IgG (Proteintech, China); PRAP antibodies (Proteintech, China) and GAPDH antibodies (Proteintech, China). Human and mouse PTX3 ELISA kits (Boster Biological Technology, China); IKK-16, and Jingzhaotoxin-III (Tocris Bioscience, USA), rhPTX3 and siPTX3 (Guangzhou Ribobio, China) and Dimethyl sulfoxide (Beyotime Biotechnology, China).

### IC_50_ and Cell Viability Assay

Inhibitory concentration value (IC_50_) of rBmK AGAP was evaluated using 3-(4-5-dimethylhiazol-2-yl)-2, 5-diphenyltetrazolium bromide (MTT) assay. MCF-7 and MDA-MB-231 cells were seeded in 96-well plates at a density of 1 × 10^4^ cells per well and incubated at 37°C overnight. The cells were then treated with different concentrations of rBmK AGAP (0, 5, 10, 15, 20, 25, 30, 35, 40, 45, 50, 55, and 60 μM) and incubated in a humidified atmosphere of 5% CO_2_ at 37°C for 24 h 0.9% saline was added to the untreated cells as the control groups. Then, 20 μL of MTT stock solution (5 mg/mL) was added to each well and cells incubated for an additional 4 h at 37°C. The MTT solution was then discarded, and 100 μL of dimethyl sulfoxide (DMSO) solution was added into each well and incubated for 30 min in the dark to dissolve the insoluble formazan crystals. Optical density (OD) was measured at a test wavelength of 590 nm and a reference wavelength of 650 nm using an enzyme-linked immunosorbent assay (ELISA) multi-well plate reader (BioTek Instruments, Winooski, USA).

The effect of rhPTX3 on cells viability was determined using the MTT assay. MCF-7 and MDA-MB-231 cells were seeded in 24-well plate at a density of 2 × 10^4^ cells per well and incubated at 37°C for 24 h. The cells were then treated with different concentrations of rhPTX3 (0, 50, 100, and 150 μM). The rest of the procedure was similar to the steps as described in IC_50_ and cell viability Assay above. To calculate percentage cell viability, OD values were used in the formula:

$$\begin{array}{l} \%\text{ of cell viability =}\frac{OD\;valueofexperimentalsample\;\left(treatedcells\right)}{\text{OD value of experimental control }\left(\text{untreated cells}\right)}\;\times \text{100} \end{array}$$

### Extreme Limiting Dilution Sphere Formation Assay

The procedure used for the limiting dilution assay was the same as previously described (31–33). Single cell suspensions of MCF-7 or MDA-MB-231 cells were obtained by passing cells through a 40 μm filter. Cells were then seeded at a density of 1,000/well to 1 cell/well in a 96-well ultralow attached plate with medium supplemented with 2% B-27, 20 ng/ml bFGF, and 20 ng/ml EGF at a final volume of 200 μL per well and cultured at 37°C in 5% CO_2_ for 14 days. Only spheroid bigger than 50 μm in size were included in the analysis. Spheroid formation with rBmK AGAP (30 μM) treated cells or untreated cells were analyzed in all wells.

### Sphere Formation Assay

Sphere formation was performed in ultralow attachment plates (Corning, USA) with medium supplemented with 2% B-27, 20 ng/ml bFGF, and 20 ng/ml EGF. The MCF-7 and MDA-MB-231 cells were plated at a density of 500 cells per well and cultured at 37°C in 5% CO_2_. Cells were then treated with different concentrations of BmK AGAP (0, 15, 30, and 60 μM). After 14 days, spheres <50 μm in diameter were counted at 40x magnification under a microscope (Olympus BX51, Japan). Sphere formation efficiency (SFE) = Number of spheres per 500 cells.

### Colony Formation Assay

Colony formation assay was performed with 500 cells seeded onto a 35 mm dish in a medium supplemented with 2% B-27, 20 ng/ml bFGF, and 20 ng/ml EGF. Cells were treated with different concentrations of BmK AGAP (0, 15, 30, and 60 μM). Surviving colonies of MCF-7 and MDA-MB-231 cells were counted with crystal violet staining after culturing for 14 days.

### Total RNA Extraction, cDNA Synthesis, and Quantitative Polymerase Chain Reaction (qPCR)

Quantitative Polymerase Chain Reaction (qPCR) was used to measure mRNA level. Total RNAs were extracted using Trizol (Takara, Japan) according to the manufacturer's protocol. RNA was reverse transcribed into cDNA using PrimeScriptTMRT reagent kit (TransGen Biotech, China), and then qPCR was performed using TransStart TipTop Green qPCR SuperMix (TransGen Biotech, China) according to the manufacturer's instructions. The sequences of the primers used were: PTX3: 5′-CATCCAGTGAGACCAATGAG-3′ (F), 5′-GTAGCCGCCAGTTCACCATT-3 (R); Nanog: 5′-CCT GTG ATT TGT GGG CCT GA-3′ (F), 5′-CTC TGC AGA AGT GGG TTG TTT G-3′ (R); Sox2: 5′-GTG AGC GCC CTG CAG TAC AA-3′ (F), 5′-GCG AGT AGG ACA TGC TGT AGG TG-3′ (R); Oct4: 5′-GCA GAT CAG CCA CAT CGC CC-3′ (F), 5′-GCC CAG AGT GGT GAC GGA GA-3′ (R); N-cadherin: 5′-AAA GAA CGC CAG GCC AAA C-3′(F), 5′-GGC ATC AGG CTC CAC AGT GT-3′(R); E-cadherin: 5′-CAA CGA CCC AAC CCA AGAA-3′(F), 5′-CCG AAG AAA CAG CAA GAG CA-3′(R); GAPDH: 5′-GCA CCG TCA AGG CTG AGA AC-′3(F), 5′-TGG TGA AGA CGC CAG TGGA-3′ (R). The thermal conditions for qPCR assay in applied biosystems stepOne™ Real-Time PCR thermal cycler (SN-271004043, Thermo Fisher Scientific, USA) were: cycle 1: 95°C for 10 min, cycle 2 (x 40): 95°C for 10 s and 58°C for 45 s. Data were normalized to GAPDH, and relative quantities were calculated using the 2^−ΔΔ*Ct*^ method. Triplicate independent experiments were carried out.

### ELISA

Human PTX3 ELISA Kit (Boster Biological Technology, China) was used to measure PTX3 secretion in supernatant samples from MCF-7 and MDA-MB-231 cells as described elsewhere (34).

### Transwell Migration and Invasion Assays

Transwell migration assay was used to determine the migration potential of breast cancer cells. MCF-7 and MDA-MBA-231 cells (5 × 10^4^) cells were pretreated with different concentrations of rBmK AGAP (0, 15, 30, or 60 μM) for 48 h and re-suspended in culture medium with the same concentrations of rBmK AGAP and seeded onto the uncoated membrane in the upper chamber of the transwell (24-well millicell cell culture insert, 12 mm diameter, 8 μm pores; Merck KGaA, #P18P01250, China). DMEM or DMEM/F12 supplemented with 10% FBS was used as an attractant in the lower chamber. After incubation for 24 h, non-migrated cells in the upper chamber were swabbed off, and cells that had migrated through the membrane were fixed with 4% paraformaldehyde and stained with 1% crystal violet. Images of stained cells were captured with a microscope (Olympus, USA), and cells in five random fields at 10 × magnification were counted.

For the invasion assay, the inner chambers of the transwell plate were coated with ECM gel (Sigma, USA) and incubated at 37°C for 1 h to produce an artificial basement membrane. The ECM gel was diluted in ice-cold serum-free DMEM to a final concentration of 2 mg/ml before used. The rest of the procedure was similar to the steps as described in the migration assay above. Both migration and invasion assays were performed concurrently.

### Western Blot Analysis

For experimental procedures, cells in the log phase of growth were harvested and washed twice with ice-cold PBS and harvested in CEB lysis buffer (Invitrogen, Life Technologies, and Grand Island, NY, 14072). Protein was quantitated using Pierce BCA Protein Assay Reagent Kit (Pierce Biotechnology, Rockford IL, USA) as per manufacturer's protocol. After protein extraction from cells and tumor tissues (9g), an equal amount (20 μg/well) of proteins were separated by 12% SDS-PAGE and transferred onto nitrocellulose (NC) membrane guided by a pre-stained protein molecular weight ladder in a wet transfer system (Beijing Liuyi Biotechnology, China). The membranes were then blocked with 5% fat-free milk in TBST at room temperature for 1 h and probed with specific primary antibodies against PTX3, Sox2, Oct4, N-cadherin, Snai1, E-cadherin, Nav 1.5, NF-κB, p65/NF-κB, p-p65, IκBα, IKKα, TNF-α, β-catenin, GSK3-β, pGSK3-β, PRAP, and GAPDH at 4°C overnight. This was followed by incubation with appropriate species-specific secondary antibodies at room temperature for 1 h. Antibody binding was detected with an enhanced chemiluminescence kit (ECL, Amersham, UK) and detected in a ChemoDocTM XRS + Imager system (Bio-Rad, USA). Relative quantities were analyzed with Image Lab software v4.0.1 (Bio-Rad, USA).

### Treatment and siRNA Transfection

MCF-7 and MDA-MB-231 cells were cultured in six-well plates in DMEM/F12 or high glucose DMEM, supplemented with 10% fetal bovine serum. At 24 h after plating, the complete medium was replaced with serum-free medium. At 80–90% cell confluence, cells were treated with 100 ng/ml of recombinant human PTX3 (rhPTX3), and for the specific down-regulation of PTX3, the MCF-7, and MDA-MB-231 cells were transiently transfected with 100 nM of PTX3-specific small interfering RNA (siPTX3) using Dharma FECT as per the manufacturer's protocol. The target siRNA sequences (GenePharma, Shanghai) against PTX3 were 5′-GCACAAAGAGGAAUCCAUATT-3′and negative control or scramble was 5′-UUCUCCGAACGUGUCACGUTT-3′. For specific down-regulation of β-catenin, cells were transiently transfected with 100 nM siβ-catenin using Dharma FECT. The target siRNA sequence against β-catenin (Guangzhou Ribobio, China) was 5′-GCTGAAACATGCAGTTGTA-3′, and negative control/ scramble was 5′-TTCTCCGAACGTGTCACGT-3′. After 6 h the serum-free medium was replaced with complete medium and cultured at 37°C in 5% CO_2_. After 48 h of transfection, the cells were collected for further experiments.

### Nuclear and Cytoplasmic Fractionation

Cytoplasmic Extraction Reagent kit (#78833, Thermo Fisher Scientific, USA) was used to perform the nuclear and cytoplasmic fractionation according to the manufacturer's protocol.

### Immunofluorescent Staining

Human breast cancer cells MCF-7 and MDA-MB-231 (2.5 × 10^5^) were cultured on glass coverslips, and after 48 h of rBmK AGAP treatment, cells were fixed with 4% paraformaldehyde for 30 min after washing with PBS. Cells were then permeabilized with 0.1% Triton X-100 for 5 min and blocked with complete serum for 30 min at 37°C. The MCF-7 and MDA-MB-231 cells were incubated with primary antibodies of interest at 4°C overnight followed by a FITC-conjugated goat anti-mouse IgM or TRITC-conjugated goat anti-rabbit IgG (Sigma-Aldrich, China) for 1 h at room temperature. The cells on the coverslips were then incubated with DAPI for 10 min at room temperature. Images were obtained under a fluorescence microscope (Olympus BX83, Japan).

### Xenograft Tumor Mouse Model

The experimental animals were provided by the Specific Pathogen Free (SPF) Animal Facility of Dalian Medical University. All experimental animal procedures were approved by the Animal Ethics Committee of the Dalian Medical University, P.R. China. Female nude BALB/c mice between the ages of 6–8 weeks old and weighing between 18 and 22 g were maintained under sterile conditions during the entire experimental period. The mice were housed in standard transparent plastic cages under 12/12 h light-dark cycle regime and were provided free access to food and water. MDA-MB-231 cells (5 × 10^6^) suspended in 0.2 ml PBS were injected subcutaneously at the right flank, and after seven days, the mice were randomly assigned to one of three groups (*n* = 7/group). An equal concentration of rBmK AGAP 1 mg/kg or 0.5 mg/kg of body weight diluted in equal volume (100 μL) of 0.9% saline was injected intraperitoneally for 20 days at 48 h interval. Mice treated with 0.9% saline only served as control (untreated). The tumor dimensions were measured every 2 days using a digital caliper (#03000002, GuangLu, China). The tumor volume was calculated according to the formula: Volume = 1/2 Length × Width^2^. On day 20, mice were euthanized, and breast tumor masses were weighed and snap-frozen in liquid nitrogen for further experiments.

### Immunohistochemical Staining

Immunohistochemistry was performed on paraffin-embedded sections of the excised human breast tumor tissues collected from breast cancer patients and visible tumors removed from mice. Serial sections (4 μm each) were prepared, deparaffinized in xylene and rehydrated in graded alcohol. After microwaving for 20 min in citrate buffer to expose antigens, the slides were washed with PBS and incubated in 3% H_2_O_2_ for 10 min at room temperature to block endogenous peroxidase activity. Non-specific binding was blocked with goat serum at room temperature for 30 min before overnight incubation at 4°C with primary antibodies of interest. After extensive washing with PBS, slides were incubated with biotinylated secondary antibody for 45 min at room temperature. The slides were then incubated with a streptavidin-peroxidase complex. The signal was visualized with DAB (3, 3′-diaminobenzidine), and the slides were briefly counterstained with hematoxylin. Yellowish brown stain indicated positive for the antigen of interest. Images of stained tissue slides were captured using a microscope (Olympus BX51, Japan).

### Statistical Analysis

Each assay was performed three times. All statistical analyses were carried out using the GraphPad Prism v 5.01 (GraphPad Software, La Jolla, CA, US). All values are depicted as a mean ± standard deviation and considered significant if *p* < 0.05. The Student's *t*-test was used for comparisons between groups, and one-way ANOVA was used for comparisons of three or more groups.

## Results

### PTX3 Overly Expressed in Breast Cancer Cells and Tissues

**Figure 1:**
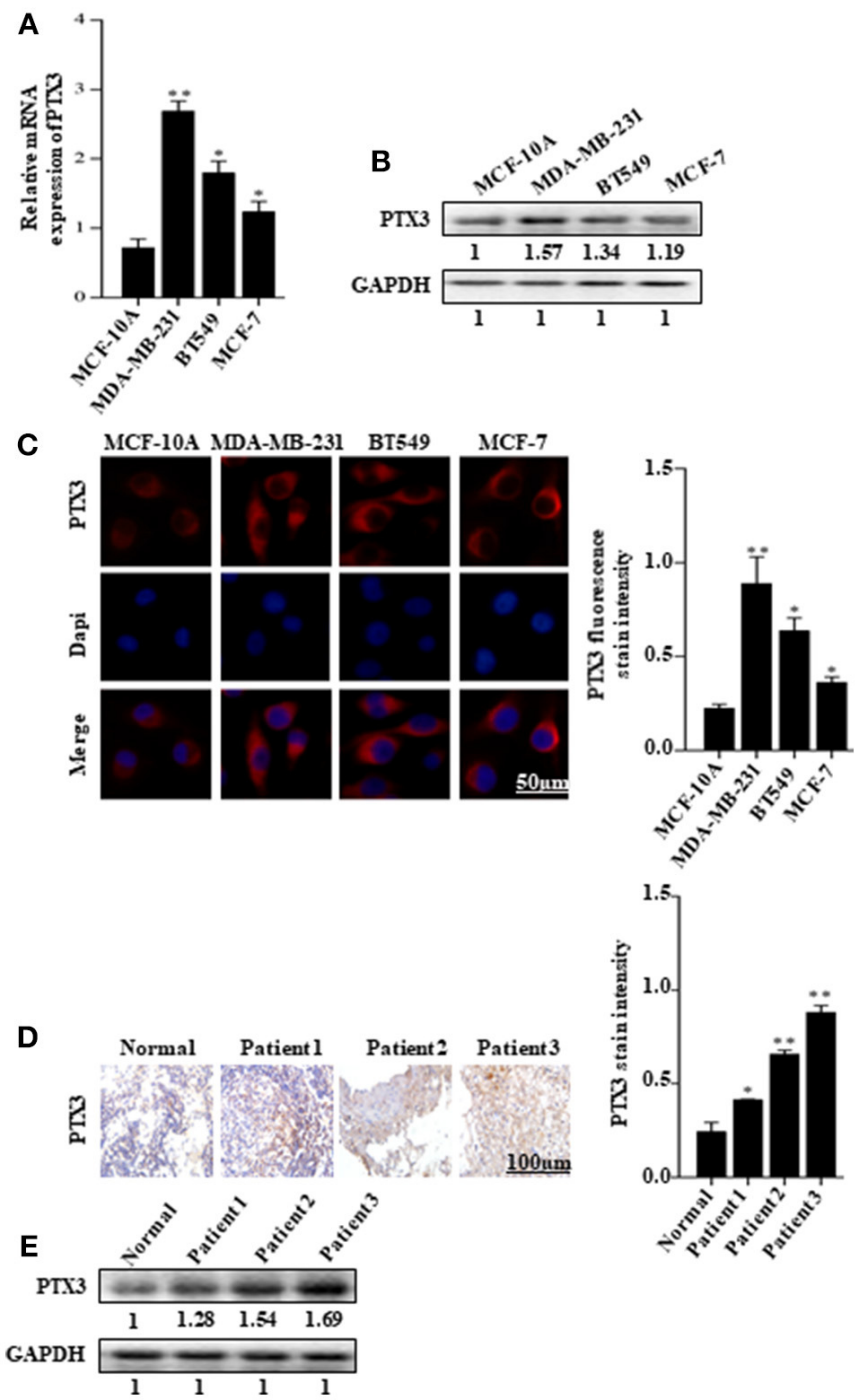
PTX3 expression in breast cancer cells and tissues. **(A)** Assessment of PTX3 gene expression relative to GAPDH in MCF-10A, MDA-MB-231, BT549, and MCF-7 cells by qPCR. **(B)** PTX3 protein expression in MCF-10A, MDA-MB-231, BT549, and MCF-7 cells. Total protein from cell lysates was subjected to 12% SDS-PAGE and analyzed by western blotting with antibodies against PTX3. GAPDH was used as loading control. **(C)** Representative micrographs of PTX3 expression in MCF-10A, MDA-MB-231, BT549, and MCF-7 cells. The cells were stained with anti-PTX3 antibodies (red) and DAPI (blue) and observed under a fluorescence microscope (bar = 50 μm; magnification, 400x). **(D)** PTX3 expression in human breast cancer tissues (patient 1, first-grade tumor, molecular subtype HER2; patient 2, second-grade tumor, molecular subtype BLBC; and patient 3, third-grade tumor, molecular subtype BLBC) examined by immunohistochemical staining. (Scale bars = 100 μm; magnification, 200x). **(E)** PTX3 expression in human breast cancer tissues and normal breast tissue. Tissues were homogenized and equal amount of proteins were subjected to SDS-PAGE and analyzed by western blotting with antibodies against PTX3. GAPDH was used as an internal control. The data are reported as mean ± SEM of three independent experiments. ^*^Indicates statistical significance, *P* < 0.05; ^**^P < 0.01.

The expression of PTX3 is high in several malignancies, including breast cancer. It is high in basal-like breast cancer (BLBC) compared with non-basal-like breast cancer (HER2, Lum A, or Lum B subgroups) (35). In this present study, to investigate the effect of BmK AGAP on breast cancer cells, we first examined PTX3 expression in MCF-10A, MDA-MB-231, BT549, and MCF-7 cells by qPCR, western blot, and immunofluorescence staining. The qPCR and western blot results showed a significant increase in PTX3 expression in MDA-MB-231 and BT549 cells as compared with MCF-10A cells. The MCF-7 cells also showed a significant increase in PTX3 expression as compared with MCF-10A cells (Figures 1A,B). Immunofluorescence staining showed similar increased PTX3 expression in MDA-MB-23, BT549, and MCF-7 cells compared with MCF-10A cells (Figure 1C). We further examined PTX3 expression in breast cancer tissues of patients: patient one (first-grade tumor, molecular subtype HER2); patient two (second-grade tumor, molecular subtype BLBC); and patient three (third-grade tumor, molecular subtype BLBC). The results showed a similar significantly increased PTX3 expression in breast cancer tissues as the tumor advanced in stages compared with the normal breast tissues (Figures 1D,E). These findings support the evidence that PTX3 expression is high in breast cancer patients.

### PTX3 Expression in Breast Cancer Cells Is Associated With Stem-Like Features and Epithelial-Mesenchymal Transition

**Figure 2:**
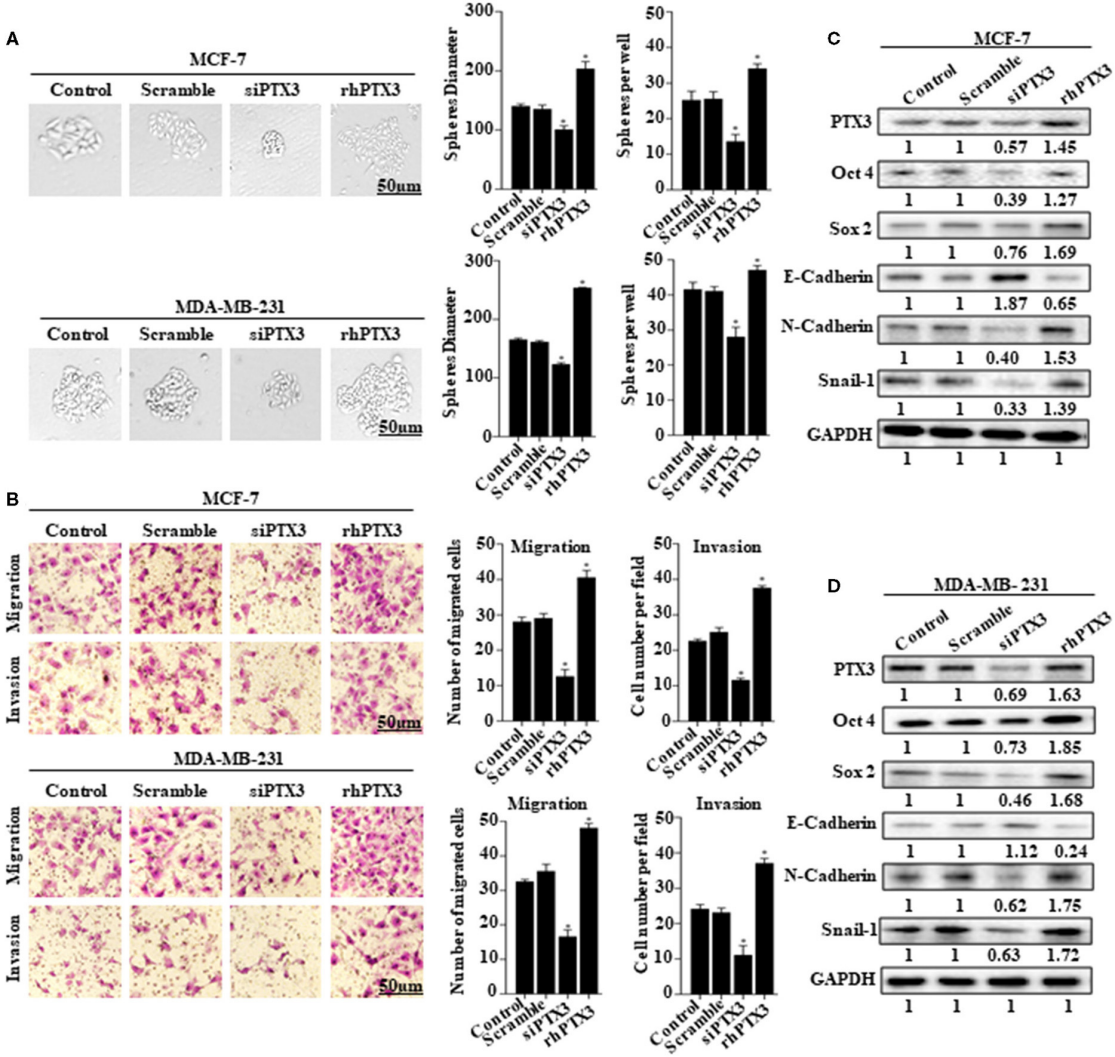
PTX3 expression in breast cancer cells is associated with stem-like features and epithelial-mesenchymal transition. **(A)** Tumorsphere formation of MCF-7 and MDA-MB-231 cells. MCF-7 and MDA-MB-231 cells were treated with siPTX3 or rhPTX3 for 14 days, and tumor spheres expansion were analyzed at 40x magnification under a microscope (bar = 50 μm; magnification, 400x). **(B)** PTX3 promotes cell migration and invasion in breast cancer. MCF-7 and MDA-MB-231 cells were treated with either siPTX3 or rhPTX3. The migration and invasion abilities of the cells were examined by migration and invasion assay (Transwell assay). **(C,D)** Effect of PTX3 on stem-like features and epithelial-mesenchymal transition markers. siPTX3 or rhPTX3-treated MDA-MB-231 and MCF-7 cells were lysed and subjected to 12% SDS-PAGE and analyzed by western blotting with antibodies against PTX3, Oct4, Sox2, E-cadherin, N-cadherin, and Snail. GAPDH was used as an internal control. The data was statistically significant at ^*^P < 0.05; ^**^P < 0.01; and ^***^P < 0.001 as compared to control. Data are represented as mean ± SEM of three independent experiments.

The role of PTX3 overexpression in breast cancer remains unclear. To determine whether PTX3 expression in breast cancer cells is associated with stem-like properties and epithelial-mesenchymal transition, we either down-regulated PTX3 expression in MCF-7 and MDA-MB-231 cells using siPTX3 or treated the cells with rhPTX3. Sphere formation assay showed a significant decrease in tumor-sphere size and number by siPTX3, while there was an increase in tumor-sphere size and number by rhPTX3 treatment compared with control (Figure 2A). Transwell assay also indicated siPTX3 to significantly decrease migration and invasion ability of MCF-7 and MDA-MB-231 cells while rhPTX3 enhanced such properties of the cells when compared with control (Figure 2B). Western blot confirmed the successful down-regulation of PTX3 in both breast cancer cells which correlated with decreased expression levels of Oct4, Sox2, and N-cadherin but increased expression of E-cadherin compared with control. rhPTX3 addition, however, stimulated increased expression levels of Oct4, Sox2, N-cadherin, and decreased expression of E-cadherin compared to control (Figures 2C,D). These data, therefore, suggest that PTX3 expression in breast cancer cells may be associated with stemness, epithelial-mesenchymal transition, and higher metastatic potential.

### BmK AGAP Inhibits Stemness of Breast Cancer Cells

**Figure 3:**
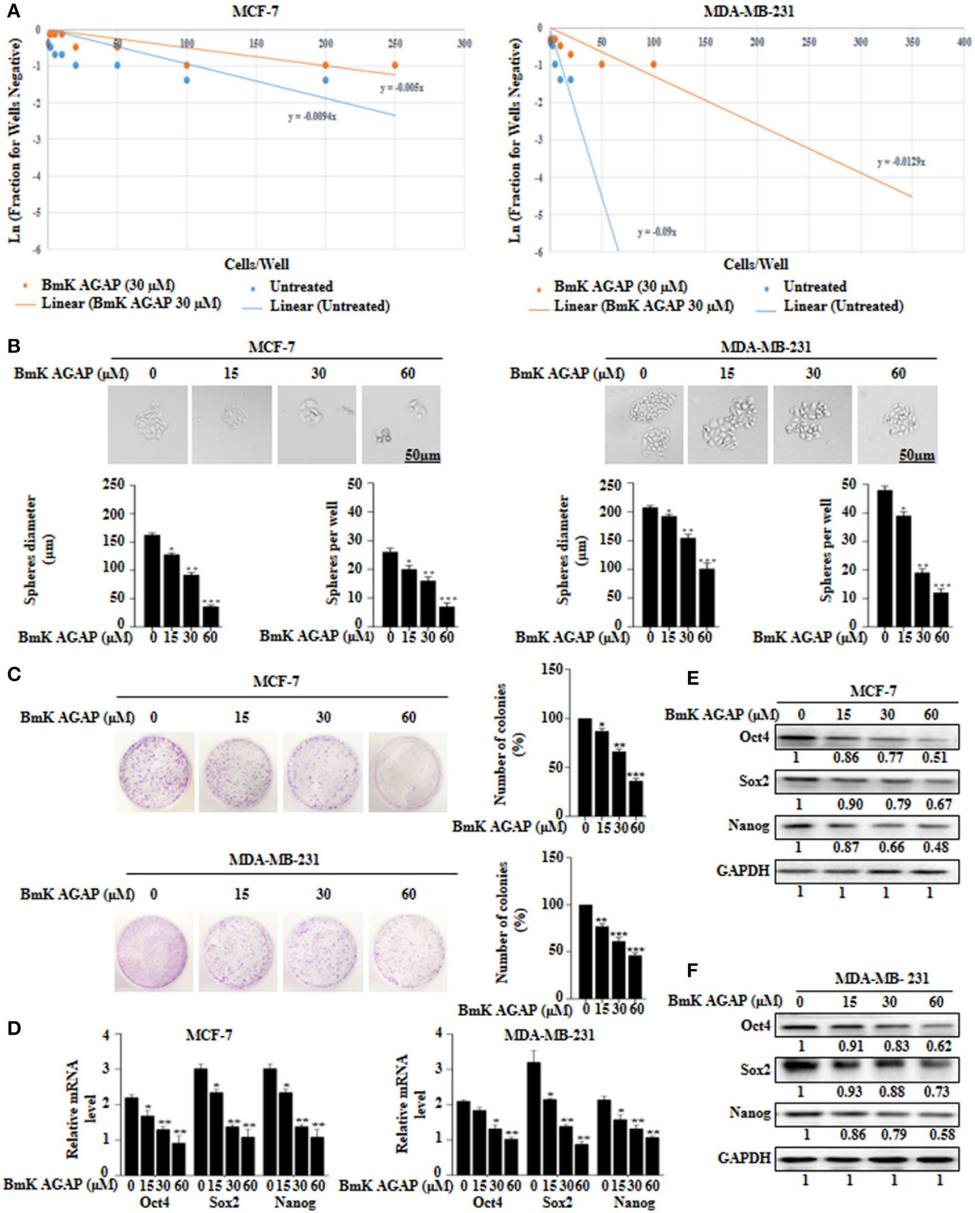
BmK AGAP inhibits stemness of breast cancer cells. **(A)** Extreme sphere limiting dilution analysis comparing stem-cell frequency in primary spheres between untreated and BmK AGAP treated MCF-7 and MDA-MB-231 cells. The cells were seeded at a density of 1,000 cells/well to 1 cell/well and cultured for 14 days. Linear (Untreated) and Linear (BmK AGAP 30 μM) indicate best fit line for linear regression analysis [Ln (1-P) = mD; where y is Ln (1-P) and x is D, (32)]. **(B)** rBmK AGAP impairs sphere formation of MCF-7 and MDA-MB-231 breast cancer cells. MCF-7 and MDA-MB-231 cells were plated at a density of 500 cells per well and treated with different concentrations of rBmK AGAP (0, 15, 30, and 60 μM) for 14 days. Spheres <50 μm in diameter were counted at 40x magnification under a light microscope. **(C)** rBmK AGAP suppresses clonal expansion of abilities of MCF-7 and MDA-MB-231 cells. MCF-7 and MDA-MB-231 cells were plated at a density of 500 cells in 35 mm dish and treated with different concentrations of rBmK AGAP and cultured for 14 days. Surviving colonies were stained with crystal violet, observed, and counted under a light microscope. **(D)** rBmK AGAP inhibits stemness of MCF-7 and MDA-MB-231 cells. MCF-7 and MDA-MB-231 cells were treated with different concentrations of rBmK AGAP. The gene expression levels of Oct4, Sox2, Nanog, and GAPDH (internal control) were analyzed by qPCR. **(E,F)** Protein expression assessment of stemness markers following rBmK AGAP treatment of MCF-7 and MDA-MB-231 cells by western blot with antibodies against Oct4, Sox2, and Nanog. (Bar = 50 μm; magnification, 400x). GAPDH was used as an internal control. The data was statistically significant at ^*^P < 0.05; ^**^P < 0.01; and ^***^P < 0.001 as compared to untreated cells. The data correspond to the mean ± SEM of three independent experiments.

To determine the effects of BmK AGAP on stem-like features in breast cancer cells, we first performed limiting dilution assay to determine the capacity of clonal expansion of MCF-7 and MDA-MB-231 cells in the presence or absence of rBmK AGAP (Figure 3A). Sphere forming frequency for the untreated vs. rBmK AGAP treated MCF-7 cells indicated 1 in 106 cells and 1 in 200 cells, respectively, whereas the sphere forming frequency for untreated vs. rBmK AGAP treated MDA-MD-231 cells indicated 1 in 11 cells and 1 in 77 cells, respectively. We then treated MCF-7 and MDA-MB-231 cells with different concentrations of rBmK AGAP for sphere and colony formation assays. Sphere formation assay showed decreased tumor-sphere size and number in both breast cancer cells following rBmK AGAP treatment compared with untreated (Figure 3B). Colony formation assay also showed both breast cancer cells to exhibit decreased colony counts following rBmK AGAP treatment (Figure 3C). Transcription factors Oct4, Sox2, and Nanog, play essential roles in maintaining the pluripotency of embryonic stem cells (36, 37). We performed qPCR and Western blot analyses to determine the expression of Oct4, Sox2, and Nanog after rBmK AGAP treatment. qPCR data showed a decrease in Oct4, Sox2, and Nanog expressions in MCF-7 and MDA-MB-231 cells in a dose-dependent manner compared with untreated cells (Figure 3D). Consistently, Western blot analysis showed similar decrease in Oct4, Sox2, and Nanog expressions as compared with untreated cells (Figures 3E,F). These results suggested that BmK AGAP may inhibit breast cancer cell stemness.

### BmK AGAP Inhibits Epithelial-Mesenchymal Transition of Breast Cancer Cells

**Figure 4:**
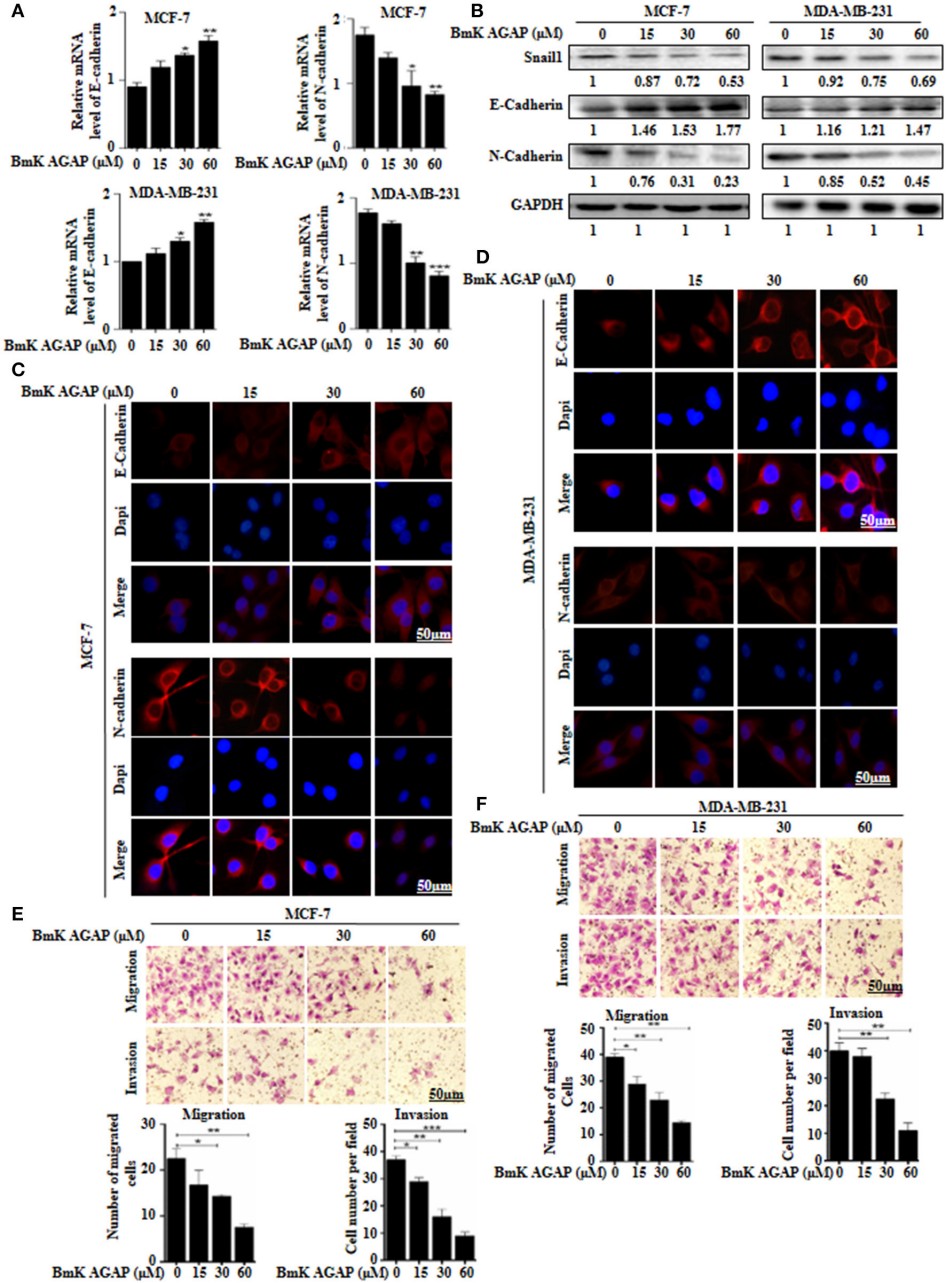
BmK AGAP inhibits epithelial-mesenchymal transition (EMT) of breast cancer cells. **(A)** Relative gene expression of N-cadherin and E-cadherin in MCF-7 and MDA-MB-231 cells treated with different concentrations of rBmK AGAP. GAPDH served as internal control. **(B)** Protein expression levels of Snail1, N-cadherin, and E-cadherin following rBmK AGAP treatment of MCF-7 and MDA-MB-231 cells. The cells were treated for 48 h, lysed, subjected to SDS-PAGE and analyzed by western blotting with antibodies against Snail1, N-cadherin, and E-cadherin. **(C,D)** Immunofluorescence assessment of cadherin and N-cadherin in MCF-7 and MDA-MB-231 cells following rBmK AGAP treatment. MCF-7 and MDA-MB-231 cells were stained with anti- E-cadherin and N-cadherin antibodies (red) following rBmK AGAP treatment and were observed under the fluorescence microscopy. DAPI was used for nuclear staining (bar = 50 μm; magnification, 400x). **(E,F)** rBmK AGAP inhibits the migration and invasion potentials of MCF-7 and MDA-MB-231 cells. MCF-7 and MDA-MB-231 cells were treated with different concentrations of rBmK AGAP and examined by migration and invasion assay. The data was statistically significant at ^*^P < 0.05; ^**^P < 0.01; and ^***^P < 0.001 as compared to untreated cells. Data are represented as mean ± SEM of three independent experiments.

To examine whether BmK AGAP has any effect on epithelial-mesenchymal transition in breast cancer cells, we treated MCF-7 and MDA-MB-231 cells with rBmK AGAP at different concentrations. We then performed qPCR, Western blot, and immunofluorescence staining to investigate the expression of epithelial marker E-cadherin and mesenchymal marker N-cadherin. mRNA expressions of E-cadherin significantly increased, and that of N-cadherin decreased following treatment with rBmK AGAP as compared with untreated cells (Figure 4A). Western blot data consistently showed that rBmK AGAP significantly increased the expression of E-cadherin in a dose-dependent manner but decreased the expression of N-cadherin and snail1 (Figure 4B). Immunofluorescence assessment also showed similar increased E-cadherin expression and decreased N-cadherin expression following rBmK AGAP treatment (Figures 4C,D). We further determined the effects of BmK AGAP on breast cancer cell migration and invasion using the transwell assay. rBmK AGAP significantly inhibited the migration and invasion ability of MCF-7 and MDA-MB-231 cells in a dose-dependent manner (Figures 4E,F). Thus, the scorpion peptide rBmK AGAP might have the ability to inhibit epithelial-mesenchymal transition in breast cancer.

### BmK AGAP Reduces the Expression of PTX3 in Breast Cancer Cells and Suppressed Cell Proliferation

**Figure 5:**
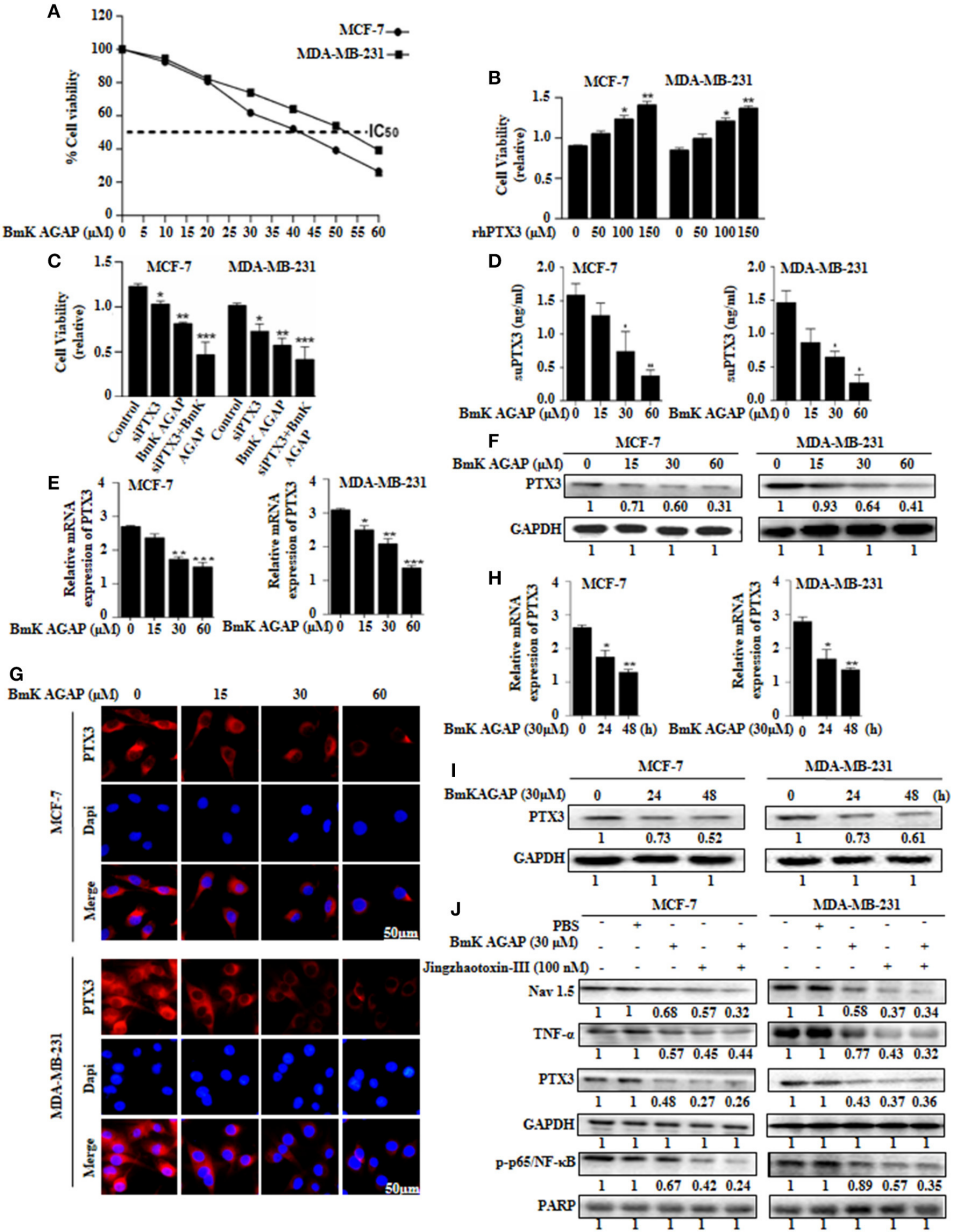
BmK AGAP suppresses the expression of PTX3 in breast cancer cells. **(A)** IC50 values of rBmK AGAP for MCF-7 and MDA-MB-231 cells. The cells were treated with different concentrations of rBmKAGAP for 24 h; cell viability was measured by MTT assay. **(B)** rhPTX3 promotes breast cancer cell survival. MCF-7 and MDA-MB-231 cells were treated with different concentration of rhPTX3 and the effect of PTX3 on cells viability examined by cell viability assay. **(C)** siRNA inhibition of PTX3 and/or rBmK AGAP treatment suppresses cell viability of breast cancer cells. MCF-7 and MDA-MB-231 cells were treated with rBmK AGAP or siPTX3 or both, and their effect on cell viability was examined by MTT assay. **(D)** rBmK AGAP suppresses PTX3 secretion. MCF-7 and MDA-MB-231 cells were treated with different concentrations of rBmK AGAP for 48 h. SecretedPTX3 in supernatant samples were measured using ELISA. **(E)** Relative gene expression of PTX3 following rBmK AGAP treatment. Cells were treated with different concentrations of rBmK AGAP for 48 h, and the expression of PTX3 and GAPDH (internal control) were analyzed by qPCR. **(F)** PTX3 protein expression following rBmK AGAP treatment of MDA-MB-231 and MCF-7 cells. rBmK AGAP treated cells were lysed and subjected to 12% SDS-PAGE and analyzed by western blotting with antibodies against PTX3. **(G)** PTX3 assessment by immunofluorescence. rBmK AGAP treated cells were stained with anti-PTX3 antibodies (red) and DAPI (blue) and observed under a fluorescence microscopy (bar = 50 μm; magnification, 400x). rBmK AGAP suppresses PTX3 expression in a time-dependent manner. MDA-MB-231 and MCF-7 cells were treated with rBmK AGAP (30 μM) for 0, 24, and 48 h and the gene **(H)** and protein **(I)** expression levels of PTX3 was examined by qPCR and western blot, respectively. GAPDH served as internal control. **(J)** rBmK AGAP (30 μM) or Jingzhaotoxin-III (100 μM) suppresses the expression of Nav 1.5, p65/NF-κB, TNF-α, and PTX3 in MCF-7 and MDA-MB-231 cells as analyzed by western blotting. GAPDH was used as an internal control. The data was statistically significant at ^*^P < 0.05; ^**^P < 0.01; and ^***^P < 0.001 as compared to untreated cells. The data represent the mean ± SEM of three independent experiments.

Choi et al. suggested that PTX3 pathway might be an effective therapeutic target for breast cancer metastasis to bone (38). In this present study, to investigate the effects of rBmK AGAP on PTX3 expression in human breast cancer cells, we first determined the inhibitory concentration value (IC_50_) of rBmK AGAP. rBmK AGAP inhibited cell proliferation in a dose-dependent manner. Compared with untreated cells, the viability of MCF-7 and MDA-MB-231 cells decreased from 96.3 to 38.9% and 95.7 to 23.6%, respectively, following rBmK AGAP treatment ranging between 5 and 60 μM (IC_50_ = 40 μM for MCF-7 and 50 μM for MDA-MB-231 cells) for 24 h (Figure 5A). We treated MCF-7 and MDA-MB-231 cells with rhPTX3 and siPTX3 to investigate the effect of PTX3 expression on cell viability. rhPTX3 promoted proliferation in MCF-7 and MDA-MB-231 cells, while siPTX3 and rBmK AGAP inhibited cell proliferation in MCF-7 and MDA-MB-231 cells (Figures 5B,C). These data suggested that PTX3 expression may influence proliferation of breast cancer cells.

We thus treated MCF-7 and MDA-MB-231 cells with different concentrations of rBmK AGAP (0, 15, 30, and 60 μM) and investigated PTX3 expression. To demonstrate the level of PTX3 protein released from cells, enzyme-linked immunosorbent assay (ELISA) was performed on supernatant specimens. There was decreased PTX3 secretion as the concentration of rBmK AGAP increased (Figure 5D). We observed a similar dose-dependent decrease in PTX3 expression at the gene and protein levels by qPCR, western blot and immunofluorescence analyses (Figures 5E–G). In a time-course assay, MCF-7 and MDA-MB-231 cells were treated with rBmK AGAP for 24 and 48 h. PTX3 expression decreased as early as 24 h at mRNA and protein levels (Figures 5H,I). These findings revealed that BmK AGAP effectively inhibits PTX3 in breast cancer cells

Scorpion venom peptide, BmK AGAP bind to voltage-gated sodium channel (VGSC) to mediate analgesic activity (30). Nav 1.5 is overexpressed in breast cancer and is associated with tumor progression (39). To investigate the possible mechanism by which BmK AGAP suppresses PTX3 expression, we inhibited voltage-gated sodium channel using Jingzhaotoxin-III to explore the involvement of Nav 1.5, in PTX3 activation/expression, and the activation of NF-κB and tumor necrosis factor (TNF)-α as essential factors for PTX3 expression. There were decreased expressions of p-p65/NF-κB, TNF-α, and PTX3 following Nav 1.5 inhibition by Jingzhaotoxin-III or BmK AGAP treatment (Figure 5J). This evidence suggested that Nav 1.5 may play a role in PTX3 expression in breast cancer.

### Nav 1.5 Is Involved in BmK AGAP Mediated Down-Regulation of PTX3 Through NF-κB and Wnt/Beta-Catenin Signaling Pathway

**Figure 6:**
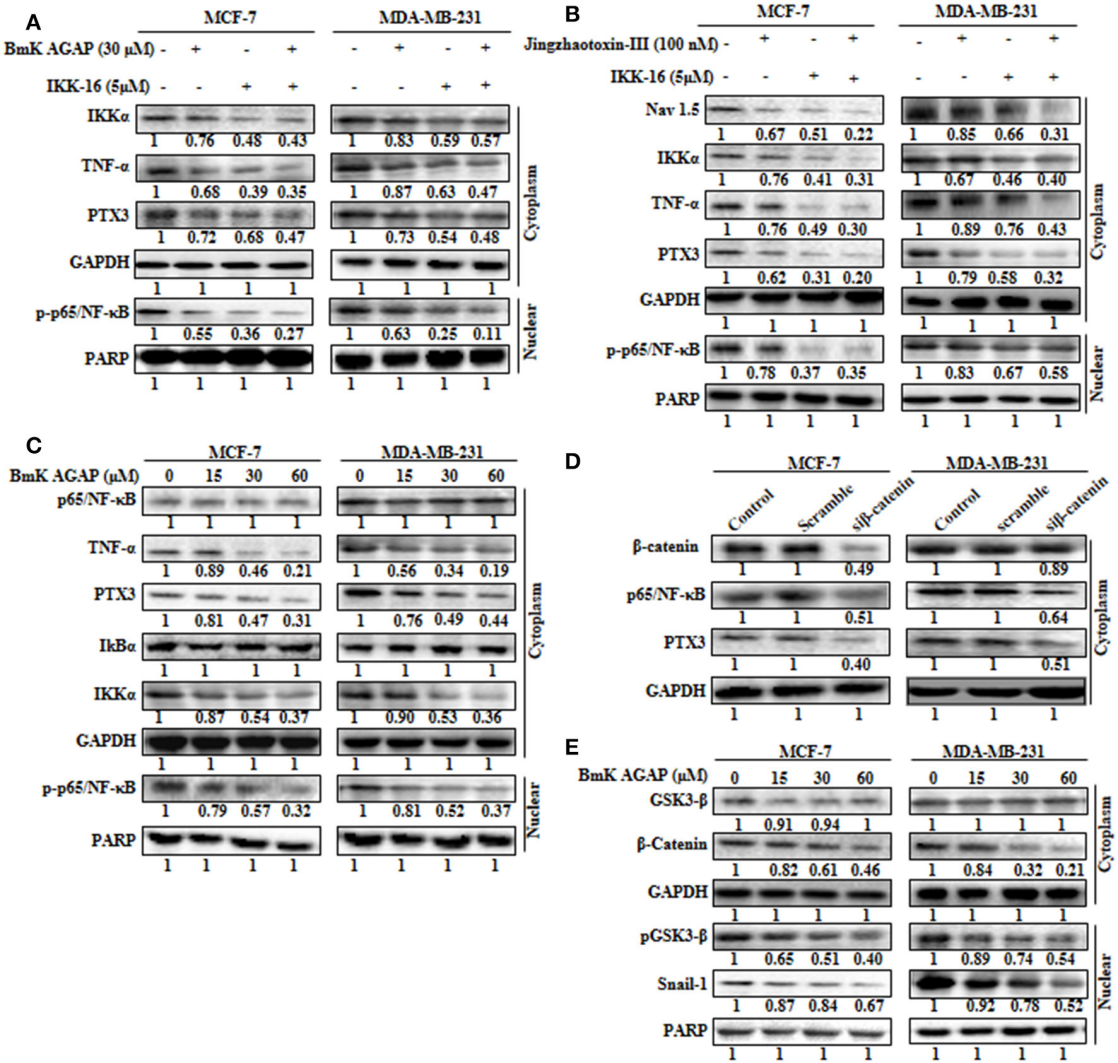
Nav 1.5 is involved in BmK AGAP mediated down-regulation of PTX3 via NF-κB and Wnt/β-catenin signaling pathway. **(A)** rBmK AGAP or IKK-16 impairs NF-κB pathway, TNF-α, and PTX3. MCF-7 and MDA-MB-231 cells were treated with either rBmK AGAP (30 μM) or IKK-16 (5 μM) for 48 h. The cells were lysed and subjected to 12% SDS-PAGE and analyzed by western blotting with antibodies against IKKα, p-p65/NF-κB, TNF-α, and PTX3. The data indicated decreased expression of IKKα, p-p65/NF-κB, TNF-α, and PTX3. **(B)** Inhibition of Nav 1.5 or IKKα suppresses PTX3, NF-κB activation, and TNF-α. Jingzhaotoxin-III or IKK-16 was used to treat MCF-7 and MDA-MB-231 cells for 48 h. The cells were then lysed and subjected to 12% SDS-PAGE and analyzed by western blotting with antibodies against Nav 1.5, IKKα, TNF-α, PTX3, and p-p65/NF-κB. The data showed decreased expression of Nav 1.5, IKKα, TNF-α, PTX3, and p-p65/NF-κB following the inhibition of Nav 1.5 or NF-κB. **(C)** rBmK AGAP suppresses NF-κB activation, PTX3, and TNF-α in breast cancer. MCF-7 and MDA-MB-231 cells were treated with different concentrations of rBmK AGAP for 48 h. Cells were lysed and subjected to SDS-PAGE and analyzed by western blotting with antibodies against p65/NF-κB, TNF-α, IKKα, IκBα, p-p65/NF-κB, and PTX3. **(D)** Impaired β-catenin pathway suppresses NF-κB and PTX3 expression. MCF-7 and MDA-MB-231 cells treated with siβ-catenin were lysed and subjected to SDS-PAGE and analyzed by western blotting with antibodies against β-catenin, PTX3, and p65/NF-κB. **(E)** rBmK AGAP suppresses β-catenin pathway. MCF-7 and MDA-MB-231 cells were treated with different concentrations of rBmK AGAP for 48 h. Cells were then lysed and subjected to SDS-PAGE and analyzed by western blotting with antibodies against β-catenin, GSK3-β, Snail 1, and pGSK3-β. GAPDH and PARP were used as internal controls. The data was statistically significant at *P* < 0.05 compared to untreated cells. The data correspond to the mean ± SEM of three independent experiments.

The expression of PTX3 is said to correlate with NF-κB in breast cancer. It is regulated by the activation of NF-κB signaling (6). Thus, inactivation of NF-κB signaling may decrease PTX3 expression in breast cancer cells by decreased p-p65/NF-κB and TNF-α. In this study, to explore the possible mechanism by which rBmK AGAP suppressed PTX3 expression, we inhibited NF-κB using IKK-16 or rBmK AGAP treatment and observed a decreased p-p65/NF-κB and TNF-α expression to correlate with decreased PTX3 expression (Figure 6A). It is reported that BmK AGAP mainly inactivates voltage-gated sodium channel to elicit its analgesic activity (40). We next inhibited Nav 1.5 using Jingzhaotoxin-III to determine the involvement of Nav1.5 in the activation of NF-κB and tumor necrosis factor (TNF)-α as essential promoters for PTX3 expression. The data confirmed a successful inhibition of Nav 1.5. We realized a decreased expression of IKKα, p-p65/NF-κB, and TNF-α in both breast cancer cells which correlated with decreased PTX3 expression (Figure 6B). Also, the inhibition of NF-κB using IKK-16 indicated similar decreased expression of p-p65/NF-κB, TNF-α, and PTX3 (Figure 6B). We next treated MDA-MB-231 and MCF-7 cells with different concentrations of rBmK AGAP to investigate further NF-κB signaling pathway. Western blot showed a decreased expression of IKKα, p-p65/NF-κB, TNF-α, and PTX3 by rBmK AGAP in a dose-dependent manner (Figure 6C). The findings suggested that Nav 1.5 may play a role in rBmK AGAP mediated down-regulation of PTX3 through NF- κB signaling pathway.

It is reported that β-catenin and NF-κB play essential roles in regulating genes in cancer cells. Studies have shown that GSK-3β, a primary substrate of β-catenin also regulates NF-κB (41). rBmK AGAP decreased the expression of β-catenin in breast cancer cells in a dose-dependent manner. To determine the association of β-catenin expression with NF-κB and PTX3 in breast cancer, β-catenin was inhibited in both cells using siβ-catenin, and then investigated the expressions of p65/NF-κB and PTX3. Successful inhibition of β-catenin by siβ-catenin indicated a decreased expressions of p65/NF-κB and PTX3 (Figure 6D). Similarly, decreased p65/NF-κB and PTX3 expressions were observed following rBmK AGAP treatment. To further investigate the effect of rBmK AGAP on the Wnt/β-catenin signaling pathway, we treated MCF-7 and MDA-MB-231 cells with different concentrations of rBmK AGAP and probed the Wnt/β-catenin signaling pathway. There were significant decreases in the expressions of β-catenin, pGSK3-β, and Snail-1 by rBmK AGAP in a dose-dependent fashion (Figure 6E). These findings suggested that rBmK AGAP down-regulate PTX3 via NF-κB and Wnt/β-catenin signaling pathway.

### BmK AGAP Inhibited the Growth of Breast Xenograft Tumors, Stem-Like Features, and Epithelial-Mesenchymal Transition in a Mouse Model

**Figure 7:**
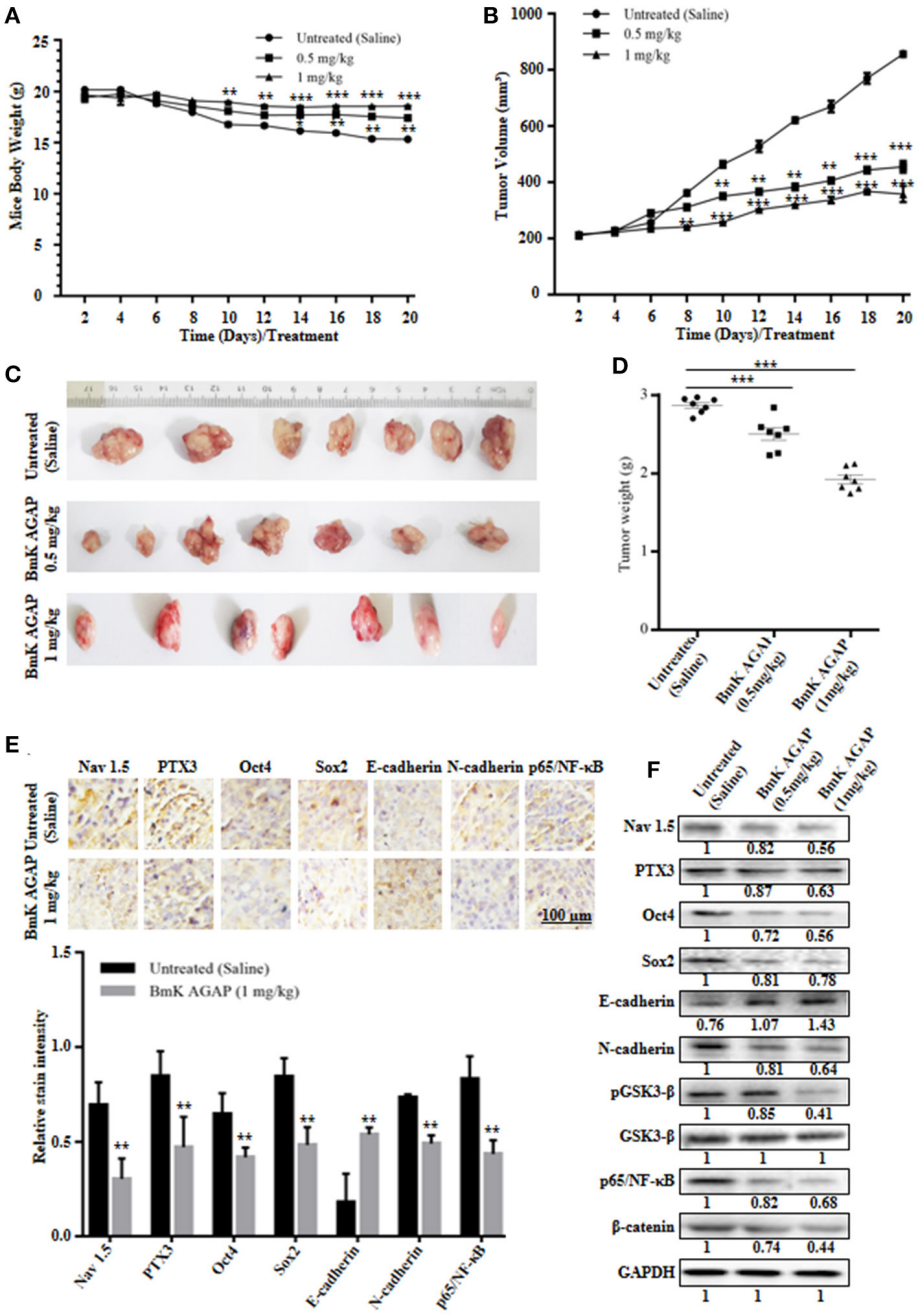
BmK AGAP inhibits the growth of breast xenograft tumors, stem-like features and epithelial-mesenchymal transition in a mouse model. **(A)** Weight changes in rBmK AGAP-treated and untreated tumor model mice. BALB/c nude mice were treated with rBmK AGAP or saline and the changes in body weight of mice bearing xenograft tumors were examined for 20 days. **(B)** Tumor volume of tumors from rBmK AGAP-treated and untreated tumor model mice. Xenograft tumor volume were calculated from measuring the length, height and width of tumors using digital caliper following rBmK AGAP treatment. **(C)** Image of excised xenograft tumors from the different treatment groups after 20 days of tumor implantation. **(D)** Quantitative analysis of excised tumor weight. Tumors excised from tumor-bearing mice sacrificed after day 20 were weighed on a digital weighting apparatus. **(E)** Immunohistochemical assessment of stemness, EMT, and inflammation markers in excised tumor tissues. Xenograft tumor tissues were stained with antibodies against Nav 1.5, PTX3, Oct4, Sox2, E-cadherin, N-cadherin, and p65/NF-κB and examined by immunohistochemical staining (Scale bars = 100 μm; magnification, 200x). **(F)** Protein expression assessment of PTX3, stemness, EMT, Wnt/β-catenin pathway and NF-κB, in excised tumors. Xenograft tumor tissues from rBmK AGAP-treated and untreated mice were lyse. Equal amount of protein samples were subjected to 12% SDS-PAGE and analyzed by western blotting with antibodies against Nav 1.5, PTX3, Oct4, Sox2, E-cadherin, N-cadherin, pGSK3-β, GSK3-β, p65/NF-κB, and β-catenin. GAPDH was used as an internal control. The data was statistically significant at ^*^P < 0.05; ^**^P < 0.01; and ^***^P < 0.001 as compared to untreated group. The data represent mean ± SD of three independent experiments.

MCF-7 human breast cancer cells were used to establish a mouse xenograft tumor model to confirm the *in vitro* observations. We investigated the role of rBmK AGAP on stem-like features, epithelial-mesenchymal transition and tumor growth *in vivo*. Body weight of mice, tumor volume, and tumor weight were analyzed in untreated, and rBmK AGAP treated mice. There was decreased tumor volume and weight in the xenograft mice treated with rBmK AGAP compared to untreated mice (Figures 7B–D). Also, there was decreased body weight of the untreated group as compared to rBmK AGAP treated groups (Figure 7A). PTX3 expression, stem-like features and epithelial-mesenchymal transition markers in xenograft tumors were analyzed. PTX3 expression in tumors decreased in rBmK AGAP treated mice. Additionally, decreased expressions of N-cadherin, Snail-1, Oct4, Sox2, β-catenin, pGSK3-β, Nav 1.5, and p65/NF-κB but E-cadherin and GSK3-β were observed in rBmK AGAP-treated mice (Figure 7F). Tissue Immunohistochemical staining for Nav 1.5, PTX3, Oct4, Sox2, E-cadherin, N-cadherin, and p65/NF-κB (Figure 7E) corroborated with observations made from western blot analyses of the same proteins.

## Discussion

The present study was designed to test the hypothesis that scorpion venom analgesic peptide, BmK AGAP inhibits cancer stemness and epithelial-mesenchymal transition by down-regulating PTX3 expression in breast cancer. The following observations emerged: the study findings support the evidence that PTX3 expression is high in breast cancer patients; PTX3 expression is associated with cancer stemness and epithelial-mesenchymal transition in breast cancer; BmK AGAP down-regulate PTX3 expression in breast cancer in a dose-dependent manner *in vitro* and *in vivo* and inhibits stemness and epithelial-mesenchymal transition. The data indicated that Nav 1.5 may play a role in BmK AGAP mediated down-regulation of PTX3 via NF-κB and Wnt/beta-catenin signaling pathway.

Most cancer patients experience pain, and that has a major impact on their daily life. It is reported that about 15% of cancer patients with pain fail to experience acceptable pain relief with the current conventional treatments (42, 43). Effective therapeutic approaches for cancer and its related pain are extremely limited, showing the need for new therapeutic interventions. Morphine and fentanyl are often used in combination with some antitumor drugs to treat cancer patients. However, some reports indicate that opioids promote cancer cell stemness, epithelial-mesenchymal transition, and chemoresistance. Yang et al. (8) reported that fentanyl promotes cancer cell stemness and epithelial-mesenchymal transition in breast cancer. Other studies have also reported that morphine promotes cancer cell stemness, epithelial-mesenchymal transition, and drug resistance in breast cancer (7, 8). These emerging pieces of evidence, therefore, supports the concept that effective therapeutic interventions for cancer-related pain are critically needed. A new purified scorpion peptide, BmK AGAP proved to have analgesic activity may present as an effective therapeutic agent for cancer and pain management. Studies have reported that BmK AGAP inhibited proliferation, migration and induced apoptosis in SW480 and SHG-44 cancer cells (40, 44). It was also reported that the analgesic peptide inhibited the migration and invasion of HepG2 cells (45). In the present study, we employed MCF-7, a differentiated breast cancer cell line that express estrogen receptor, and MDA-MB-231, a poorly differentiated triple- negative breast cancer cell line, as models and found that the scorpion analgesic peptide BmK AGAP, could effectively inhibit breast cancer cell stemness and epithelial-mesenchymal transition both *in vitro* and *in vivo*. The *in vivo* data also showed that BmK AGAP suppressed xenograft tumor volume and weight (Figure 7). Thus, making it a potential therapeutic agent against breast cancer and related pain.

Inflammation is an imperative protective reaction in a variety of physiological conditions. Notwithstanding, it contributes to the pathogenesis of several diseases, including cancer (46, 47). Within the tumor microenvironment, inflammatory reactions play a major role in the proliferation and survival of malignant cells, angiogenesis, stemness, metastasis, immune surveillance, and response to hormones, and chemotherapy (48). Cancer cell stemness, invasive capability, and metastasis potentials have been perceived to increase in the presence of inflammatory cytokines, Tumor necrosis factor (TNF)-α, and transcription factor NF-κB (49). Therapeutic strategies targeting inflammatory response in cancer may be an effective approach to cancer and pain treatment. Non-steroidal anti-inflammatory drugs including aspirin have been found to inhibit TNF-α, NF-κB, and Wnt/β-catenin signaling pathway and to reduce the risk of cancer (50, 51). Also, the perioperative use of non-steroidal anti-inflammatory drugs decreased recurrence and mortality in patients undergoing curative resection for cancer (52). PTX3 is a modulator of the inflammation process and plays a role in the activation of complement and the regulation of inflammation (53). PTX3 exerts critical roles in the recruitment of leukocyte into inflamed tissues and clearance of apoptotic cells (54). The activation of PTX3 plays a critical role in tumor-associated inflammation and chemoresistance during breast cancer treatment (55). Basile et al. (56) reported that NF-κB binding site, p65/NF-κB is functionally relevant in PTX3 promoters, TNF-α. The role of NF-κB in TNF-α is crucial for the molecular mechanisms underlying the regulations of PTX3. NF-κB mediates the induction of the transcriptional activity of PTX3 promoters by TNF-α (56). Expression of PTX3 in breast cancer is high and is associated with stem-like features, epithelial-mesenchymal transition, migration, invasion, and metastasis (57). A report indicated that CEBPD activates PTX3 transcription by directly binding to its promoter region in M2 macrophages and CAFs. PTX3 is involved in CEBPD-induced acquired chemoresistance, stemness, and metastasis/invasion of cancer cells. The activation of PTX3 in M2 macrophages or CAFs promotes the growth, metastasis, and invasion of drug-resistance cancer (58). Chan et al. (57) also reported that the activation of Akt/NF-κB signaling pathway promoted PTX3 expression in head and neck squamous cell carcinomas (HNSCC). Depletion of PTX3 and inhibition of NF-κB decreased tumor cell migration and invasion. Also, epithelial-mesenchymal transition (EMT) markers, such as vimentin and MMP-3 are reduced in PTX3 depleted cells (57). Chang et al. (59) also identified PTX3 as a promoting factor that mediates EFG-induced HNSCC metastasis. EGF-induced PTX3, in turn, induces metastatic molecules, activating inflammation reaction and metastasis. EGF-induced PTX3 transcriptional activation is by the binding of C-Jun to the activator protein (AP)-1 binding site of the PTX3 promoter. PI3K/Akt and NF-κB are crucial for PTX3 activation. PI3K/Akt and NF-κB–dependent regulation of AP-1 mediates PTX3 transcriptional responses to EGF (59). In this study, we demonstrated that PTX3 expression is associated with stemness and epithelial-mesenchymal transition in breast cancer cells. We observed a decreased sphere size, cell migration and invasion in the depleted PTX3 using the siPTX3, while the rhPTX3 indicated increased sphere size, cell migration and invasion (Figure 2). The increased expression of PTX3 and its close association with cancer-related inflammation informs its clinical importance and the need for therapeutic approaches targeting PTX3 or its mediators in cancer. rBmK AGAP treatment decreased PTX3 expression *in vitro* and *in vivo* which correlated with decreased stem-like features, epithelial-mesenchymal transition, migration, and invasion of breast cancer cells. This suggests BmK AGAP activity in decreasing breast cancer cell stemness and epithelial-mesenchymal transition to target PTX3.

The transcription factor NF-κB plays an essential role in the regulation of cell growth and survival. Activation of NF-κB signaling promotes cancer cell growth and chemoresistance (60). Abnormal activation of NF-κB is associated with several cancers, including breast cancer, pancreatic cancer, colon cancer, and melanoma (6, 61). In a previous study, it was observed that NF-κB was necessary to drive PTX3 expression to regulate the propagation of stem cell-like traits in breast cancer cells (27). Here, decreased expression of PTX3 post rBmK AGAP treatment correlated with decreased IKKα, p65/NF-κB, TNF-α, and NF-κB DNA binding and transcription activity inhibiting breast cancer cell stemness and epithelial-mesenchymal transition. The inactivation of NF-κB signaling is linked to decreasing cell growth, apoptosis, and enhanced sensitivity to chemotherapies (62). It is reported that ion channels are associated with different stages of human cancer progression, including proliferation and migration (63, 64). A study indicated that the expression of sodium ion channel is high in metastasized human breast cancer (65). Chen et al. reported that TNF-α increases voltage-gated sodium current by the activation of the channel and also increases the expression of VGSCs through NF-κB and p38 MARK signal pathways in CNS neurons (66). In prostate cancer, VGSC α-subunit, Nav 1.7 functional expression is associated with high metastatic potential (67). BmK AGAP blocks neuron transmission by binding to voltage-gated sodium channels (VGSC) at site 3 and inactivate the activated sodium channels to elicit analgesic activity (40, 68). Zhao et al. reported that BmK AGAP inhibits Nav 1.5 in SHG-14 cells and down-regulates p-AKT, pp-38, p-Erk1/2, and p-c-Jun, which gradually decreased the expression of NF-κB (40). Guo et al. also reported that the analgesic peptide up-regulated the VGSC β1 subunit to inhibit migration and invasion of HepG2 cells (45). In this present study, the role of rBmK AGAP on the expression of Nav 1.5, PTX3, and TNF-α as well as NF-κB signal transduction, were further investigated to illustrate the potential molecular mechanism. We observed a significantly decreased expression of Nav 1.5 after rBmK AGAP treatment which correlated with decreased expression of p-p65/NF-κB, TNF-α, and PTX3 in breast cancer cells. This evidence suggests that Nav 1.5 functional activities may play a role in PTX3 expression in breast cancer, through which rBmK AGAP mediate down-regulation of PTX3 via NF-κB signaling pathway.

Deng et al. (69) found that β-catenin plays a significant role as a mediator for the cross-regulation of NF-κB by GSK-3β pathway (60). We discovered that rBmK AGAP inhibited pGSK-3β, GSK-3β, and β-catenin *in vitro* and *in vivo*. The use of siβ-catenin demonstrated similar decreased NF-κB activation which correlated with decreased PTX3 level. The activation of Wnt/β-catenin signaling pathway plays an essential role in epithelial-mesenchymal transition and epithelial plasticity in normal development. Abnormal activation of the Wnt/β-catenin signaling is associated with oncogenesis (69, 70). Expression of β-catenin is associated with E-cadherin in cell-cell adhesion (71). Similarly, NF-κB expression is reported to be associated with the E-cadherin complex in epithelial cells (72). Aberrant expression of β-catenin activates the Wnt signaling pathway in breast cancer cells. Activation of Wnt/β-catenin signaling is found to correlate with cancer cell stemness and epithelial-mesenchymal transition (73–75). The abnormal activation of Wnt/β-catenin signaling induces Snail1 expression, increases vimentin and decreases E-cadherin (76–78). The findings of this study revealed decreased expression of β-catenin, Oct4, Sox2, Snail1, and the subsequent increased expression of E-cadherin by rBmK AGAP both *in vitro* and *in vivo* which to reduce breast cancer cell stemness and epithelial-mesenchymal transition.

## Conclusion

BmK AGAP may find use in inhibiting cancer cell stemness, epithelial-mesenchymal transition, migration and invasion, and for treating cancer-related pain. High expression of PTX3 increased stem-like features (Oct4, Sox2, and Nanog), decreased the expression of E-cadherin and increased other epithelial-mesenchymal transition markers. Thus, PTX3 may be a potential target for regulating cancer cell stemness and epithelial-mesenchymal transition, and a plausible therapeutic target or strategy. BmK AGAP inhibition of breast cancer cell stemness, epithelial-mesenchymal transition, migration, and invasion may be an effective therapeutic approach for breast cancer and related pain.

## Availability of data

All datasets use and analyzed during the present study are available from the corresponding author on reasonable request.

## Author Contributions

SK and Q-PW conceived and designed the study with inputs from QY. Q-PW and QY were responsible for the supervision and coordination of the project. SK and BA performed most of the experiments. SK and BA led the data analysis with inputs from QY, ZL, YC, TA, ND, EK, LO, and TZ. The first draft of the manuscript was written by SK, and then Q-PW, QY, TA, ND, EK, and LO contributed to revising and reviewing the manuscript. All authors read and approved the final manuscript before submission.

### Conflict of Interest Statement

The authors declare that the research was conducted in the absence of any commercial or financial relationships that could be construed as a potential conflict of interest.
