# Corrigendum: Scorpion Venom Analgesic Peptide, BmK AGAP Inhibits Stemness and Epithelial-Mesenchymal Transition by Down-Regulating PTX3 in Breast Cancer

**DOI:** 10.3389/fonc.2021.639813

**Published:** 2021-06-29

**Authors:** Sylvanus Kampo, Bulbul Ahmmed, Tingting Zhou, Lawrence Owusu, Thomas Winsum Anabah, Natacha Raissa Doudou, Eugene Dogkotenge Kuugbee, Yong Cui, Zhili Lu, Qiu Yan, Qing-Ping Wen

**Affiliations:** ^1^ Department of Anesthesiology, Dalian Medical University, Dalian, China; ^2^ Department of Anesthesiology, First Affiliated Hospital of Dalian Medical University, Dalian, China; ^3^ Department of Anesthesia and Intensive Care, School of Medicine and Health Science, University for Development Studies, Tamale, Ghana; ^4^ Department of Biochemistry and Molecular Biology, Dalian Medical University, Dalian, China; ^5^ Department of Biotechnology, Dalian Medical University, Dalian, China; ^6^ Department of Radiology, Dalian Medical University, Dalian, China; ^7^ Department of Clinical Microbiology, School of Medicine and Health Science, University for Development Studies, Tamale, Ghana; ^8^ School of Life Science and Bio-pharmaceutics, Shenyang Pharmaceutical University, Shenyang, China; ^9^ Department of Ophthalmology, First Affiliated Hospital of Dalian Medical University, Dalian, China

**Keywords:** scorpion venom analgesic peptide, rBmK AGAP, stemness, epithelial-mesenchymal transition, pentraxin 3, Wnt/β-catenin signaling, transcription factor NF-κB, breast cancer

In the original article, there was a mistake in **Figure 2** as published. We acknowledge making a mistake with the western blot used for Oct 4 in **Figure 2D**. The corrected **Figure 2** appears below.

**Figure 2:**
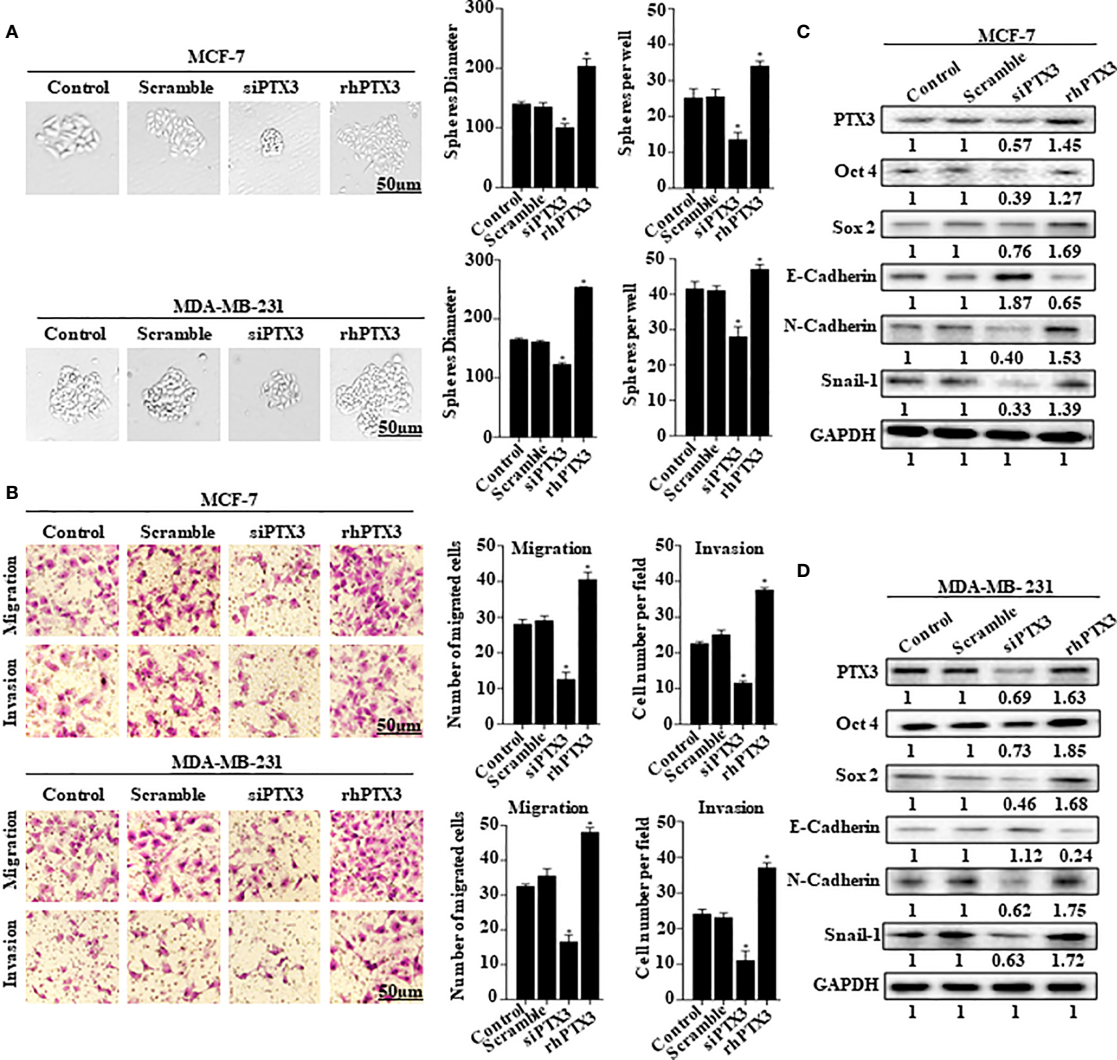
PTX3 expression in breast cancer cells is associated with stem-like features and epithelial-mesenchymal transition. **(A)** Tumorsphere formation of MCF-7 and MDA-MB-231 cells. MCF-7 and MDA-MB-231 cells were treated with siPTX3 or rhPTX3 for 14 days, and tumor spheres expansion were analyzed at 40x magnification under a microscope (bar = 50µm; magnification, 400x). **(B)** PTX3 promotes cell migration and invasion in breast cancer. MCF-7 and MDA-MB-231 cells were treated with either siPTX3 or rhPTX3. The migration and invasion abilities of the cells were examined by migration and invasion assay (Transwell assay). **(C, D)** Effect of PTX3 on stem-like features and epithelial-mesenchymal transition markers. siPTX3 or rhPTX3-treated MDA-MB-231 and MCF-7 cells were lysed and subjected to 12% SDS-PAGE and analyzed by western blotting with antibodies against PTX3, Oct4, Sox2, E-cadherin, N-cadherin, and Snail. GAPDH was used as an internal control. The data was statistically significant at *P < 0.05 as compared to control. Data are represented as mean ± SEM of three independent experiments.

The authors apologize for this error and state that this does not change the scientific conclusions of the article in any way. The original article has been updated.

